# Genome-wide analysis of the FOXA1 transcriptional regulatory network identifies super enhancer associated LncRNAs in tamoxifen resistance

**DOI:** 10.3389/fgene.2022.992444

**Published:** 2022-09-20

**Authors:** Xiulei Zhang, Qian Zhang, Guangzhi Liu

**Affiliations:** ^1^ Department of Microbiome Laboratory, Henan Provincial People’s Hospital, Zhengzhou University, Zhengzhou, China; ^2^ Henan Provincial Key Medical Laboratory of Genetics, Henan Provincial People’s Hospital, Zhengzhou University, Zhengzhou, China

**Keywords:** breast cancer, tamoxifen resistance, FoxA1, super enhancer, ATP1A1-AS1

## Abstract

Breast cancer is the leading cause of death in female cancers, and what’s worse, tamoxifen resistance occurs in almost 30% breast cancer patients and has seriously attenuated the therapeutic effect. It is widely studied that epigenetic regulation has played important role in the development of tamoxifen resistance. FOXA1 is a pioneer transcription factor that can translate epigenetic signature into transcription regulation and also drive genome-wide enhancer reprogramming in breast cancer. However, the chromatin super enhancer landscape orchestrated by FOXA1 and the key downstream targets of the FOXA1 oncogenic network in tamoxifen resistance remain elusive. Through analyzing the FOXA1 ChIP-seq data in tamoxifen sensitive MCF7 and tamoxifen resistant MCF7/TamR cells, we show that the FOXA1 chromatin occupancy is enhanced in both the promoter and enhancer regions, and the recruitment events may be E2 dependent in both MCF7 and MCF7/TamR cells. By integratively analyzing the FOXA1 ChIP-seq data and RNA-seq data of MCF7 and MCF7/TamR cells, we find that the enhanced or reduced FOXA1 chromatin binding densities may synchronize the transcriptional activity in tamoxifen resistance. Besides, we identify 1003 super enhancer associated protein coding genes and five super enhancer associated lncRNAs (ATP1A1−AS1, CASC11, CASC15, KCTD21−AS1, LINC00885) in tamoxifen resistance. By KM survival analysis, we find that high expression level of ATP1A1−AS1 and its sense transcript ATP1A1 indicates favorable clinical outcome among the luminal endocrine treated breast cancer patients. Further coexpression analysis indicates that ATP1A1-AS1 is significantly correlated with ATP1A1, and RT-qPCR results show that they both are downregulated in MCF7/TamR cells. Our study shows that the FOXA1 transcriptional regulatory network may promote the development of tamoxifen resistance, and identifies one super enhancer associated lncRNA ATP1A1-AS1 that may work as promising biomarker or drug target in tamoxifen resistance.

## Introduction

Breast cancer has ranked highest incidence rate and mortality rate in female cancers around the world (Sung et al., 2021). More than 70% breast cancer patients are estrogen receptor positive (ERα+), thus making ERα a promising treatment target. Tamoxifen, the selective ERα modulator, has significantly reduced more than 30% mortality rate among the ERα+ breast cancer patients (Group, 2005). However, almost 30% breast cancer patients have recurred or relapsed during the tamoxifen treatment due to intrinsic or acquired resistance (Davies et al., 2013). Tamoxifen resistance has been a challenge in the treatment of ERα+ breast cancer. Previous studies demonstrate that changes in the epigenetic landscape and deregulation of transcription factors orchestrate the tamoxifen resistant phenotype (Garcia-Martinez et al., 2021). Our published work presents that the differential ERα recruitment events and the mediated differential expression profiles lead to the occurrence and development of tamoxifen resistance (Zhang et al., 2020). FOXA1 is a pioneer transcription factor that recruits ERα and other mediators to regulate the transcription, and FOXA1 always collaborates with transcription factors in the enhancer regions and translates the epigenetic signature into changes in transcription activity. What’s more, FOXA1 also facilitates the H3K27ac epigenetic reprogramming in the enhancer regions and leads to metastatic and tamoxifen resistance phenotype (Fu et al., 2019). Enhancer, a distal genomic element, is critical for transcription regulation, and the enhancer malfunction is associated with initiation, progression, and metastasis of many cancers (Lee et al., 2020). Super enhancers are stretch enhancers, where enhancers cluster together and exert more potent effect in the transcription regulation. Super enhancers are more active in the transcription regulation of oncogenes and promote cancer pathogenesis (Hnisz et al., 2013). LncRNAs are important part of noncoding transcripts in the cancerogenesis and drug resistance by pre or post transcription regulation (Li et al., 2016). In this study, we aim to present the FOXA1 chromatin binding landscape in the enhancer regions, and identify FOXA1 regulated super enhancer associated lncRNAs in tamoxifen resistance. By integratively analyzing the FOXA1 ChIP-seq and RNA-seq data of tamoxifen-sensitive (MCF7) and tamoxifen-resistant (MCF7/TamR) breast cancer cells, we show that the enhanced or reduced FOXA1 chromatin binding densities may synchronize the transcriptional activity in tamoxifen resistance, and identify one super enhancer associated lncRNAs ATP1A1−AS1 that may exert important functions in tamoxifen resistance.

## Materials and methods

### Cell culture

MCF7/TamR cells was derived from its parental cell line MCF7, and it was continuously treated with tamoxifen (1 μM) (MCE, United States) more than 1 year (Zhang et al., 2018). MCF7 and MCF7/TamR cells were authenticated through the STR profile analysis (Genewiz, China). MCF7 and MCF7/TamR cells were cultured in DMEM (BI, Israel), 10% fetal bovine serum (BI, Israel), and 1% penicillin-streptomycin (Solarbio, China), and MCF7/TamR cells were continuously cultured with 1 μM tamoxifen. MCF7/TamR cells were directly treated with tamoxifen (5 μM) for 24 h. The estradiol (E2) (MCE) treatment (10 nM, 6 h) was performed before the MCF7/TamR cells were hormone starved for 3 days.

### ChIP-seq data analysis

The FOXA1 and H3K27ac ChIP-seq data in MCF7 and MCF7/TamR were obtained from the GEO database (GSE113092). The MCF7/TamR cell line was derived and maintained by continuously treating the MCF7 cells with 100 nM tamoxifen (Cocce et al., 2019). The reads were first subject to quality control with the FastQC package, and then mapped with the bowtie2 package using the hg38 reference genome (Andrews, 2010; Langmead and Salzberg, 2012). The generated sam files were filtered, merged and reordered with the Samtools package (MAPQ≥30), and peaks were called using the MACS2 package with default parameters (Zhang et al., 2008; Li et al., 2009). The called peaks were annotated by the ChIPseeker package using the hg38 annotation genome (Yu et al., 2015). The enhancers and super enhancers were identified using the ROSE package following the default parameters, and the annotation of the enhancer and super enhancer regions were also performed with the ROSE package (Whyte et al., 2013). The −3 kb_3 kb regions around the TSS were set as the promoter regions, and the enhancer regions were identified from the FOXA1 binding peaks in MCF7 upon E2 treatment by the ROSE package. The FOXA1 and H3K27ac ChIP-seq binding density around the transcription start site (TSS) and the enhancer center (−3 kb_3 kb) were presented using the deeptools package (Ramirez et al., 2016). The differential binding peaks were identified with the Diffbind package, FDR <0.05, Fold >0 was regarded as gained peaks, and FDR <0.05, Fold <0 was regarded as lost peaks (Stark and Brown, 2011).

### RNA-seq data analysis

The RNA-seq data of MCF7 and MCF7/TamR was obtained from the GEO database (GSE106681). These 2 cell lines were the same as the cell lines for ChIP-seq (Cocce et al., 2019). The reads were first performed quality control with the FastQC package, and the low quality reads in the 5’ end were trimmed with the cutadapt package (Martin, 2011). The trimmed reads were then mapped to the hg38 genome with the hisat2 package, and the generated sam files were filtered and sorted with the Samtools package (MAPQ≥30) (Kim et al., 2015). The annotation and differential expression analysis were conducted with the HTSeq and DEseq2 package, and |logFC|≥1 and *p* < 0.05 were set as the threshold (Love et al., 2014; Anders et al., 2015). The integrative analysis of ChIP-seq and RNA-seq data of MCF7 and MCF7/TamR cells was performed by the BETA package following the default parameters (Wang et al., 2013).

### Function enrichment analysis

The GSEA and KEGG function enrichment analysis of differential binding genes of ChIP-seq data and misregulated genes of RNA-seq data in MCF7 and MCF7/TamR cells was conducted using the clusterProfiler package (Yu et al., 2012). The KEGG function enrichment analysis of the FOXA1 ChIP-seq binding genes was performed with the clusterProfiler package, and the regions were set around TSS (−3 kb_3 kb). *p*< 0.05 was set as the threshold.

### Survival analysis

The survival analysis of the candidate super enhancer associated lncRNAs was performed through the KMplot website (https://kmplot.com/), and the enrolled patients were ERα+ breast cancer and only treated with endocrine therapy (Lánczky and Gyrffy, 2021). Median was set as the cut-off, and *p* < 0.05 was regarded as significant.

### The Cancer Genome Atlas-BRCA data analysis

The clinical and transcriptome data of TCGA-BRCA were obtained from the Cancer Genome Atlas (TCGA) website. The phenotype information of TCGA-BRCA was obtained from the UCSC Xena database, and used to extract luminal TCGA-BRCA data for following analysis. The Pearson Correlation analysis between ATP1A1-AS1 and ATP1A1 was performed with cor.test function in R language using the luminal TCGA-BRCA transcriptome matrix. Cor≥0.3 and *p* < 0.05 were used as the threshold. For all data used in this work were downloaded from TCGA website and the work complied with the terms of use of TCGA website, approval and informed consent from the Clinical Research Ethics Committee were not required.

### RT-qPCR validation

Further RT-qPCR validation of the differential expression of the candidate lncRNA was conducted on the MCF7 and MCF7/TamR cells. RNA was extracted using the TRIzol regent (Vazyme, China) following the manufacturers’ protocol, and the reverse transcript reaction and quantified PCR were performed using the RT-qPCR kit (Vazyme, China). The primers were designed through the Primer3 and BLAST tool of the NCBI website, and the specificity of the primers was validated by the melt-curve analysis on the stepone plus machine (Invitrogen, United States). The reaction condition was following our previous article (Zhang et al., 2021). The sequence of the specific qPCR primers was 18S, forward-GTAACCCGTTGAACCCCATT, reverse-CCATCCAATCGGTAGTAGCG; ATP1A1-AS1, forward-GAAGTGCACAGCAACCAAGC, reverse-ACCTCTGGCACCAAACAGTG; ATP1A1, forward-GACCGAATTCCTGCTGACCT, reverse-AGTGCGATCCCCAGTGTAGA; GREB1, forward-ATGGGAAATTCTTACGCTGGAC, reverse-CACTCGGCTACCACCTTCT. The qPCR results were analyzed by ΔΔCt method, and presented using the GraphPad software. The statistical analysis was performed with two-tail paired t-test, and *p* < 0.05 was regarded as significant.

## Results

### FOXA1 chromatin occupancy in the promoter and enhancer regions

**FIGURE 1:**
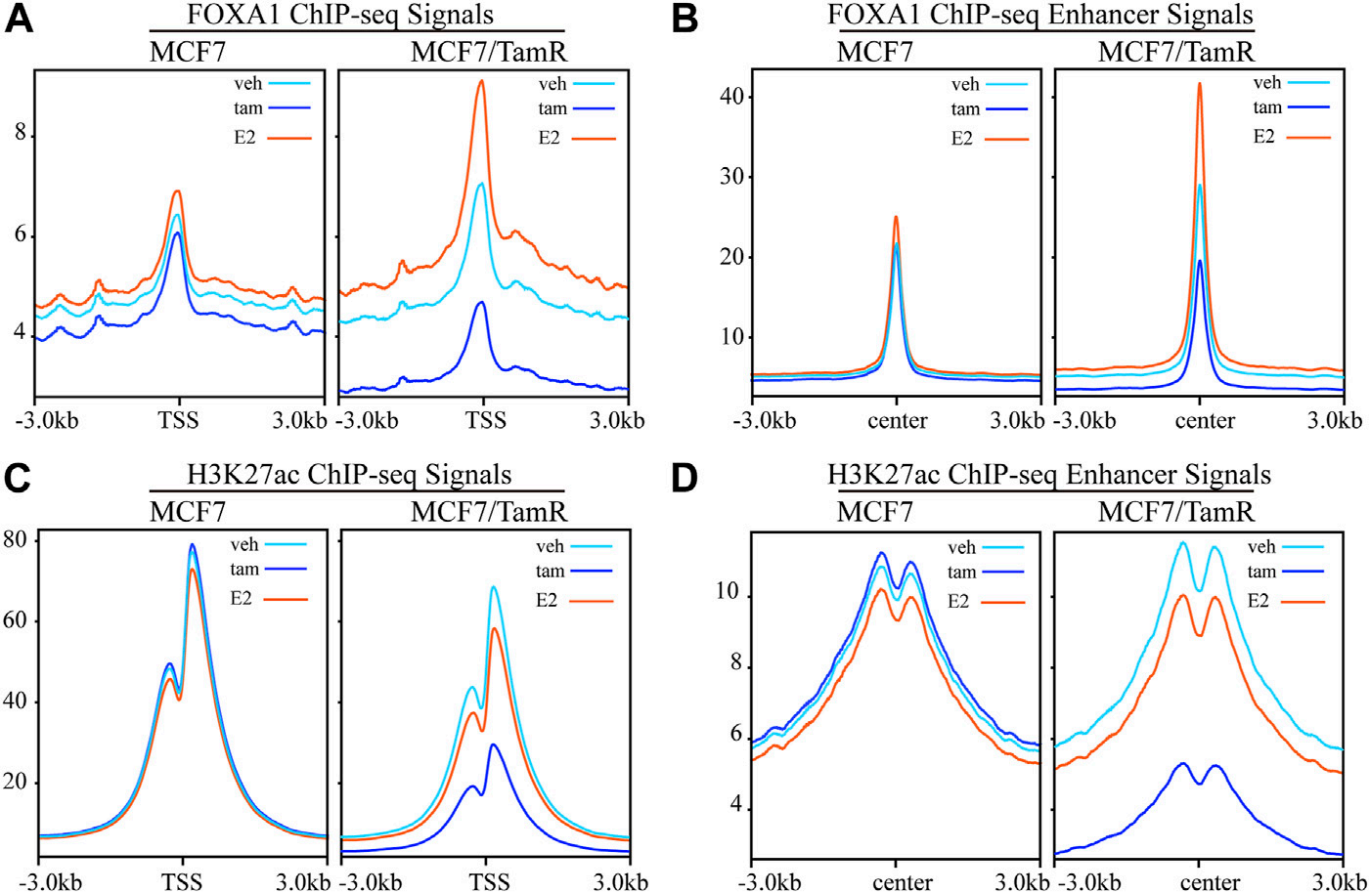
The FOXA1 and H3K27ac ChIP-seq signals in the promoter and enhancer regions. The FOXA1 ChIP-seq signals of MCF7 and MCF7/TamR cells treated with vehicle (veh), tamoxifen (tam) and estradiol (E2) around the TSS **(A)** or enhancer center **(B)**. The H3K27ac ChIP-seq signals of MCF7 and MCF7/TamR cells treated with vehicle (veh), tamoxifen (tam) and estradiol (E2) around the TSS **(C)** or enhancer center **(D)**. Light blue line, dark blue line and light red line respectively represent vehicle (veh), tamoxifen (tam) and estradiol (E2) treatment.

FOXA1 and H3K27ac play important role in the epigenetic regulation of breast cancer, including tamoxifen resistance. In order to further investigate the FOXA1 and H3K27ac genome-wide transcription regulation pattern in tamoxifen resistance, we analyze the FOXA1 and H3K27ac ChIP-seq data in MCF7 and MCF7/TamR cells. We present the FOXA1 and H3K27ac chromatin binding landscape. The chromatin binding densities of FOXA1 are elevated in both promoter and enhancer regions in MCF7/TamR cells when compared with MCF7 cells (Figures 1A,B). What’s more, the chromatin binding signals of FOXA1 in both promoter and enhancer regions are enhanced when MCF7 or MCF7/TamR cells are treated with E2, and decreased when treated with tamoxifen (Figures 1A,B). Besides, the H3K27ac ChIP-seq signals in the enhancer regions are also increased in MCF7/TamR cells when compared with MCF7 cells (Figure 1D). However, H3K27ac doesn’t show the same trend as FOXA1 when MCF7 or MCF7/TamR cells treated with E2 or tamoxifen (Figures 1C,D). FOXA1 may be a pioneer transcription factor in both promoter and enhancer regions, and also E2 dependent transcription factor in both tamoxifen sensitive and resistant cell lines.

### The differential FOXA1 chromatin binding profiles

**FIGURE 2:**
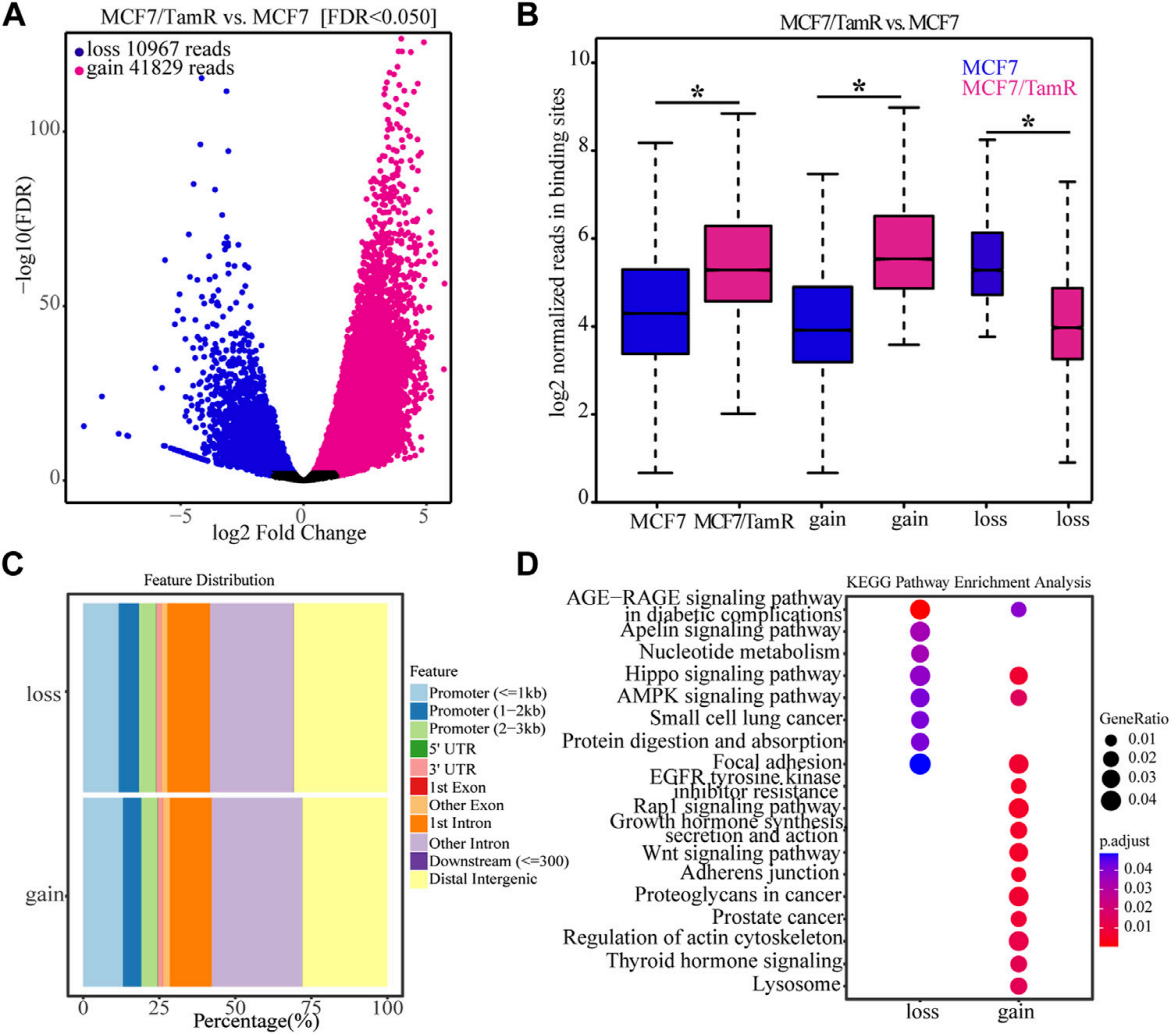
The FOXA1 gained and lost chromatin binding reads in MCF7/TamR cells. **(A)** Volcano plot of the FOXA1 gained and lost chromatin binding reads in MCF7/TamR cells, red dot represents gain and blue dot represents loss. **(B)** Box plot of the FOXA1 gained and lost chromatin binding reads in respectively MCF7 and MCF7/TamR cells, red box represents MCF7/TamR cells and blue box represents MCF7 cells. **(C)** The feature distribution of the FOXA1 gained and lost chromatin binding reads in MCF7/TamR cells. **(D)** Dot plot of the function enrichment analysis of the FOXA1 gained and lost chromatin binding reads in MCF7/TamR cells. * indicates *p* < 0.05.

For the important role of FOXA1 in the tamoxifen resistance, we further analyze the differential FOXA1 chromatin binding profiles in MCF7 and MCF7/TamR cells. We find that there are 41829 gained reads and 10967 lost reads in MCF7/TamR cells (FDR < 0.05), and the gained reads are almost four times than lost reads (Figure 2A) (Supplementary Table S1). These gained and lost reads are significantly differential between MCF7 and MCF7/TamR cells (Figure 2B), which may lead to differential FOXA1 genome-wide transcription regulation reprogramming. To further show the FOXA1 global distribution feature of the gained reads and lost reads, we analyze the FOXA1 ChIP-seq positioning, but they don’t show obviously different distribution feature (Figure 2C). We then perform the functional enrichment analysis of the gained and lost reads, and find that they are enriched in different functional categories. The gained reads in MCF7/TamR are mainly enriched in regulation of actin cytoskeleton, adherens junction, Wnt signaling pathway, Rap1 signaling pathway, and growth hormone synthesis, secretion and action. On the contrast, the lost reads are primarily enriched in nucleotide metabolism, Hippo signaling pathway, AMPK signaling pathway, protein digestion and absorption, and focal adhesion (Figure 2D). The differential FOXA1 chromatin binding profiles in MCF7 and MCF7/TamR cells may cause active or repressive signaling pathways, and further lead to the occurrence and development of tamoxifen resistance.

### FOXA1 cistromes synchronize the transcriptional activity in tamoxifen resistance

**FIGURE 3:**
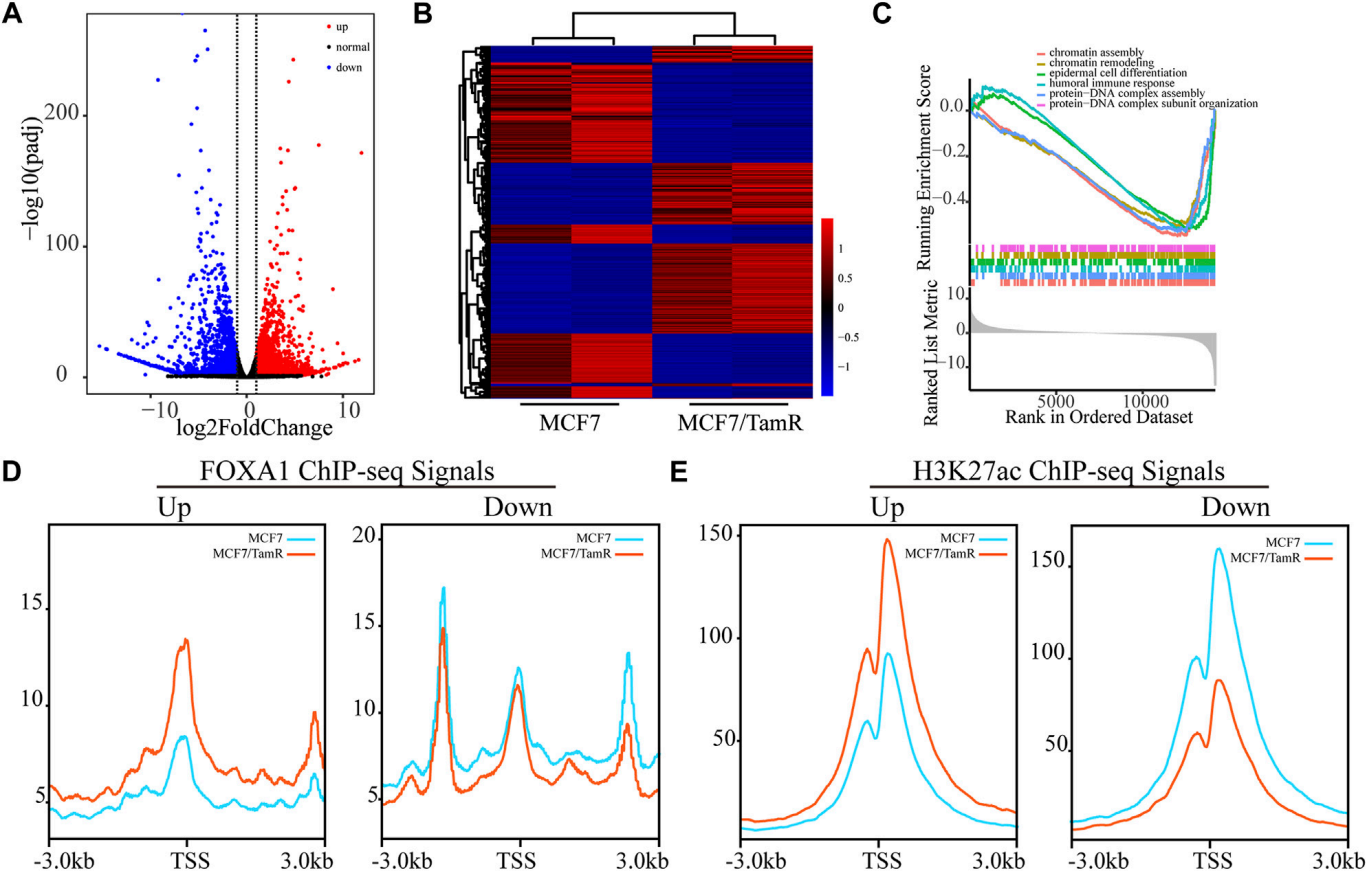
FOXA1 cistromes synchronize the transcriptional activity in tamoxifen resistance. **(A)** Volcano plot of the RNA expression profile in MCF7 and MCF7/TamR cells, red dot represents upregulated RNA, black dot represents normal RNA and blue dot represents downregulated RNA. **(B)** Heatmap of the misregulated RNA expression profile in MCF7 and MCF7/TamR cells. **(C)** GSEA analysis of the misregulated RNAs. The FOXA1 **(D)** and H3K27ac **(E)** chromatin binding densities plot of the upregulated and downregulated RNAs in MCF7 and MCF7/TamR cells, red line represents MCF7/TamR cells and the blue line represents MCF7 cells.

As FOXA1 acts as key transcription factor and its enhanced chromatin recruitments in both the promoter and enhancer regions in MCF7/TamR cells, we analyze the RNA-seq data between MCF7 and MCF7/TamR cells to investigate the FOXA1 directly altered expression profile. By differential expression analysis, we get 1970 upregulated genes and 2065 downregulated genes (FDR < 0.05, |log2Foldchange|≥1) (Figures 3A,B) (Supplementary Table S2). The GSEA analysis results show that the abnormal signaling pathways in tamoxifen resistance include chromatin assembly, chromatin remodeling, epidermal cell differentiation, humoral immune response, protein−DNA complex assembly, and protein−DNA complex subunit organization (Figure 3C). To determine whether the altered expression profile results from the changes of FOXA1 and H3K27ac chromatin binding signals, we analyze the FOXA1 and H3K27ac chromatin binding densities around the TSS of the upregulated and downregulated genes. The results show that the chromatin binding densities of FOXA1 and H3K27ac are enhanced around the TSS of the upregulated genes, and decreased around the TSS of the downregulated genes in MCF7/TamR cells (Figures 3D,E). On the contrary, the cumulative FOXA1 and H3K27ac levels around TSS of non-differentially regulated genes (−1<log2Fold change<1 & FDR > 0.05) upon resistance don’t show an obvious pattern as the differential expression genes (Supplementary Figure S1). The integrative analysis of FOXA1 and H3K27ac ChIP-seq data and the RNA-seq data in MCF7 and MCF7/TamR cells shows that FOXA1 and H3K27ac exert positive transcription regulation in the MCF7/TamR cells (Supplementary Figure S2). FOXA1 chromatin binding densities may synchronize the transcriptional activity in tamoxifen resistance, which indicates that the transcription regulation of FOXA1 and H3K27ac in MCF7/TamR may promote the occurrence and development of tamoxifen resistance.

### The super enhancer associated lncRNAs

**FIGURE 4:**
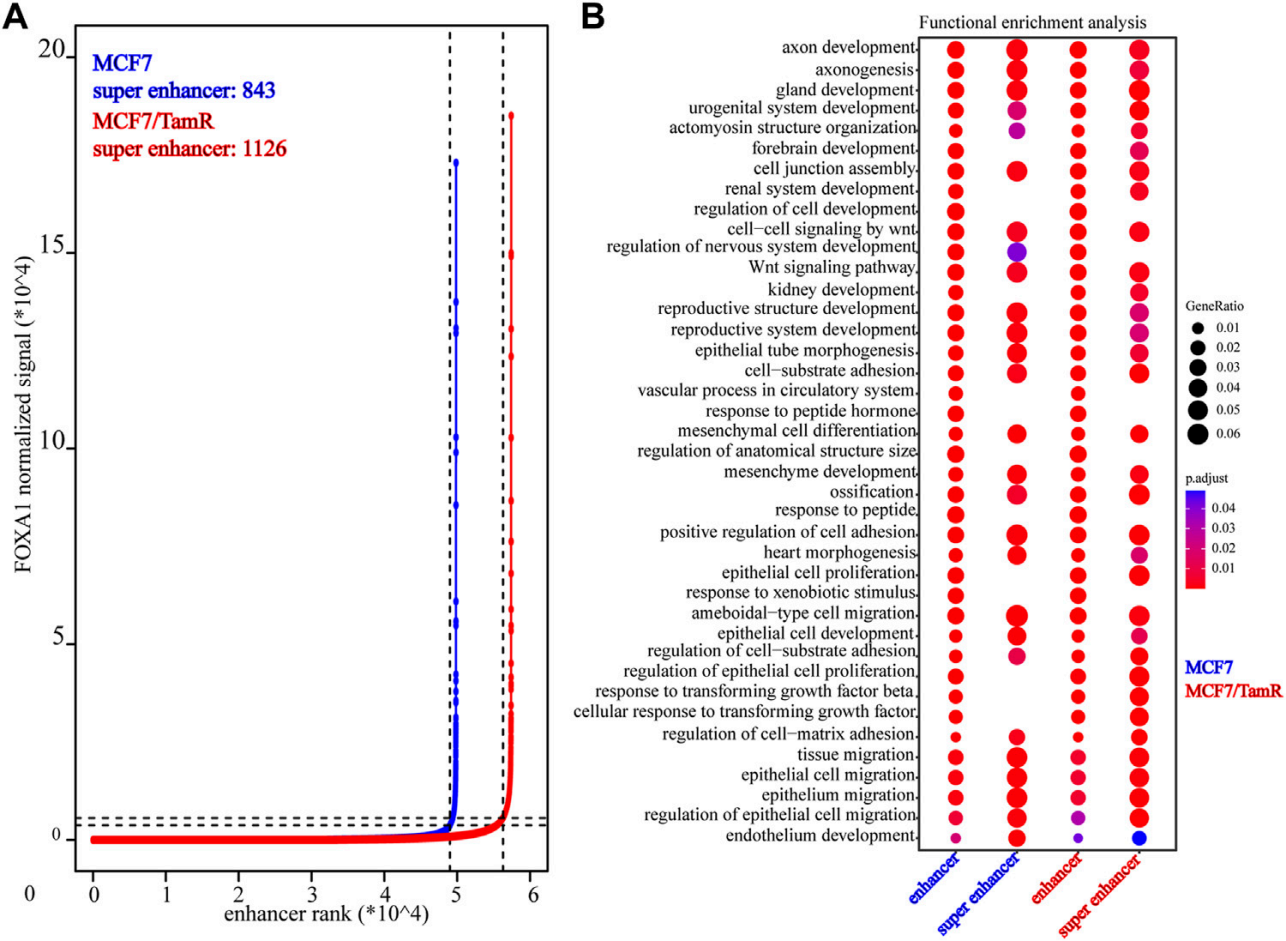
The identified super enhancers in MCF7 and MCF7/TamR cells. **(A)** Distribution of FOXA1 normalized ChIP-seq signal and the corresponding enhancer rank in MCF7 and MCF7/TamR cells, red line represents MCF7/TamR cells and the blue line represents MCF7 cells. **(B)** Dot plot of the function enrichment analysis of the identified enhancers and super enhancers in MCF7 and MCF7/TamR cells.

**FIGURE 5:**
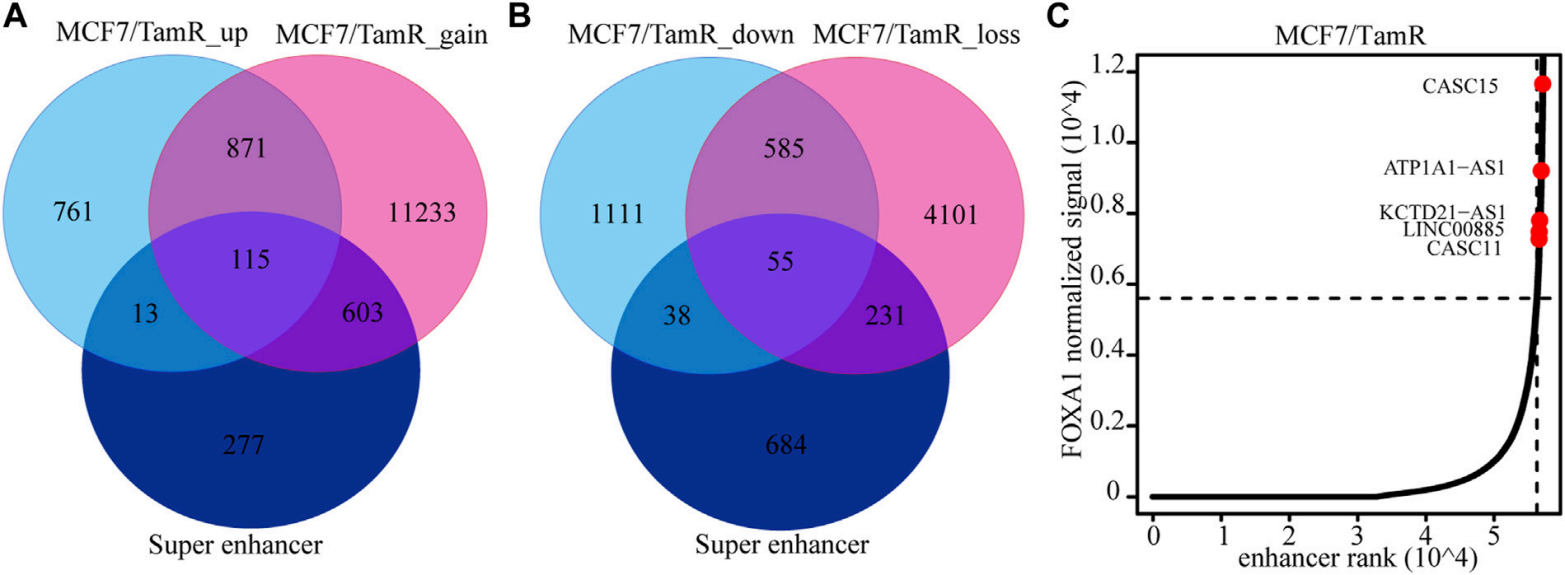
The identified super enhancer associated lncRNAs. **(A)** The venn diagram of the upregulated RNAs in MCF7/TamR, FOXA1 gained reads in MCF7/TamR and super enhancer associated RNAs. **(B)** The venn diagram of the downregulated RNAs in MCF7/TamR, FOXA1 lost reads in MCF7/TamR and super enhancer associated RNAs. **(C)** Distribution of FOXA1 normalized ChIP-seq signal and the corresponding enhancer rank in MCF7/TamR cells, and red dot represents lncRNA.

Previous studies validate the key role of FOXA1 in the enhancer and super enhancer regulation, and we find that the regulation of FOXA1 in the enhancer activity may promote tamoxifen resistance. We further analyze the super enhancer regulation of FOXA1 in MCF7 and MCF7/TamR cells. By analyzing the FOXA1 ChIP-seq data, we identify 843 and 1126 super enhancer associated genes respectively in MCF7 and MCF7/TamR cells (Figure 4A) (Supplementary Table S3). Enhancer and super enhancer are pioneer master regulator in the transcription regulation, and we further perform the functional enrichment analysis of the enhancer and super enhancer associated genes. We find that enhancer associated genes in MCF7 and MCF7/TamR cells are almost enriched in the same categories, and the super enhancer associated genes show somewhat differential (Figure 4B). The super enhancer associated genes in MCF7/TamR are mainly enriched in epithelial cell proliferation and transforming growth factor beta signaling pathways (Figure 4B). To identify the key super enhancer associated genes, we make an overlap of the upregulated genes, FOXA1 gained binding genes and the identified super enhancer associated genes (Figure 5A), and on the other hand, the other overlap of downregulated genes, FOXA1 lost binding genes and the identified super enhancer associated genes (Figure 5B). We finally get 115 upregulated super enhancer associated genes and 55 downregulated super enhancer associated genes, including five super enhancer associated lncRNAs (ATP1A1−AS1, CASC11, CASC15, KCTD21−AS1, LINC00885) (Figure 5C). These five super enhancer associated lncRNAs may play an important function in the development of tamoxifen resistance.

### The potential function of ATP1A1−AS1

**FIGURE 6:**
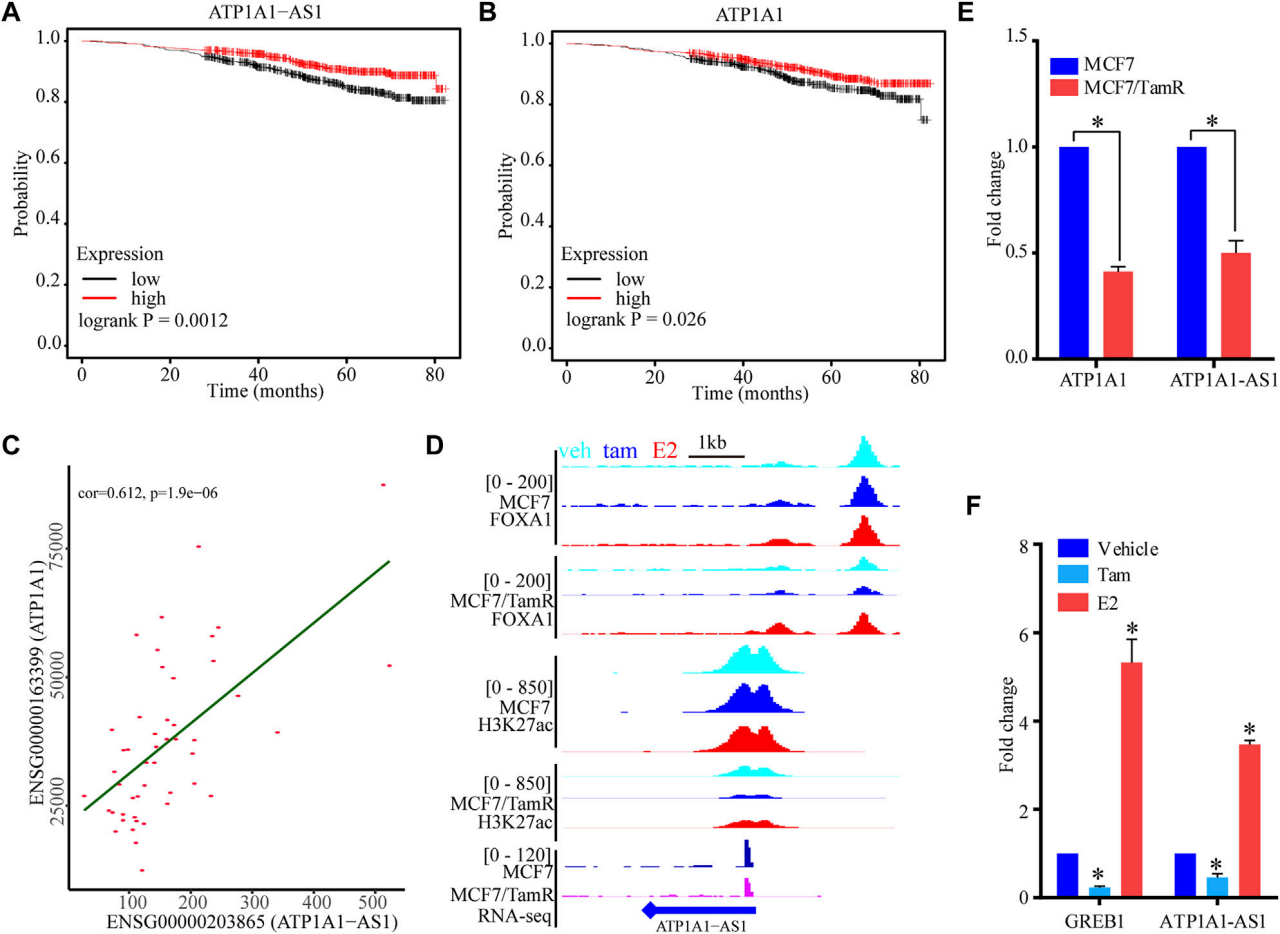
The potential function of ATP1A1-AS1. The KM survival curve of ATP1A1-AS1 **(A)** and ATP1A1 **(B)** using the luminal breast cancer treated with endocrine therapy datasets. **(C)** Coexpression analysis of ATP1A1-AS1 and ATP1A1 using the luminal TCGA-BRCA datasets. **(D)** The FOXA1 chromatin binding peak distribution of MCF7 and MCF7/TamR cells in the upstream of ATP1A1-AS1, light blue peak, dark blue peak and light red peak respectively represent vehicle (veh), tamoxifen (tam) and estradiol (E2) treatment. **(E)** Bar plot of the expression level of ATP1A1-AS1 and ATP1A1 in MCF7 and MCF7/TamR cells. **(F)** Bar plot of the expression level of ATP1A1-AS1 and GREB1 in MCF7/TamR cells upon E2 and tamoxifen treatment. * indicates *p* < 0.05.

To determine the prognosis value of these five super enhancer associated lncRNAs, we use the datasets of ERα+ breast cancer patients only treated with endocrine to perform survival analysis of these lncRNAs. The high expression level of ATP1A1-AS1 shows favorable clinical outcome, and the high expression level of its sense transcript ATP1A1 also indicates high survival probability (Figures 6A,B). The other three lncRNAs (CASC15, KCTD21−AS1, LINC00885) don’t show significant prognosis value (Supplementary Figure S3). To determine the correlationship between ATP1A1-AS1 and ATP1A1, we perform correalation analysis of them using the luminal breast cancer patient datasets from TCGA database. We find ATP1A1-AS1 is significantly positive correlated with ATP1A1, which may suggest their same prognosis value (Figure 6C). The downregulated ATP1A1-AS1 in MCF7/TamR cells may be caused by the reduced chromatin binding densities of FOXA1 and H3K27ac (Figure 6D). What’s more, the ChIP-seq data analysis results show that enhanced chromatin binding densities of FOXA1 and H3K27ac in the upstream of ATP1A1-AS1 in MCF7/TamR cells when treated with E2, and decreased chromatin binding signals in the upstream of ATP1A1-AS1 in MCF7/TamR cells when treated with tamoxifen (Figure 6D). However, this result is not obvious in MCF7 cells (Figure 6D). In the RT-qPCR results, ATP1A1-AS1 and ATP1A1 is significantly downregulated in MCF7/TamR cells, and ATP1A1-AS1 is up-regulated by E2 and down-regulated by tamoxifen (Figures 6E,F). Thus, ATP1A1-AS1 is a super enhancer associated lncRNA regulated by FOXA1 and H3K27ac in an E2 dependent manner in the tamoxifen resistant cells, and may play an important role in the development of tamoxifen resistance.

## Discussion

The majority of breast cancer patients are ERα+, and tamoxifen has been an effective adjuvant treatment. But almost one third ERα+ breast cancer patients have recurred or relapsed due to tamoxifen resistance. Tamoxifen resistance has seriously reduced the therapeutic efficacy of ERα+ breast cancer patients (Yao et al., 2020). Tamoxifen resistance can be driven by specific expression profiles that result from transcription regulation reprogramming by master transcription factors. We previously reported that the dynamic ERα chromatin binding landscape in tamoxifen resistance may mediate the specific transcription activity that leads to the refractory phenotypes (Zhang et al., 2020). FOXA1 is a pioneer transcription factor that can facilitate the ERα and other cofactors to the specific genomic regions to initiate transcription activity (Hurtado et al., 2011). What’s more, FOXA1 always collaborates with transcription factors in the enhancer regions and translates the epigenetic signature into changes in transcription activity (Fu et al., 2019). By analyzing the FOXA1 ChIP-seq data of MCF7 and MCF7/TamR, we present the FOXA1 differential chromatin recruitment events in tamoxifen resistance. We find the enhanced FOXA1 chromatin binding densities in both promoter and enhancer regions in MCF7/TamR than MCF7. There are 41829 gained FOXA1 binding reads and 10967 lost FOXA1 binding reads, and the gained reads are almost four times of lost reads. To further illustrate the active and depressed signaling pathways that result from FOXA1 gained or lost chromatin binding events, we perform the function enrichment analysis of the gained reads and lost read. We find that the active signaling pathways are adherens junction, Wnt signaling pathway, Rap1 signaling pathway, and growth hormone synthesis, and the depressed signaling pathways are Hippo signaling pathway, AMPK signaling pathway, and focal adhesion. Among these signaling pathways, activation of AMPK signaling can promote the ERRα/PGC-1β-MCAD/CPT-1 and increase fatty acid oxidation, which lead to the autophagy of tamoxifen resistant breast cancer cells (Duan et al., 2021). Whereas, activation of AMPK signaling also inhibits mTOR/HIF-1α signaling and the glycolysis-dependent metabolism in tamoxifen resistant breast cancer cells (Woo, et al., 2015). Activated Wnt signaling have been demonstrated to be associated with acquired tamoxifen resistance, and maintain the stem cell-like properties of ER + breast cancer (Loh et al., 2013; Ham et al., 2022). Besides, the enhanced or decreased FOXA1 chromatin binding densities are altered by respectively the E2 or tamoxifen treatment in both MCF7 and MCF7/TamR cells, which indicate that the FOXA1 chromatin occupancy is E2 dependent. Published articles report that not only FOXA1 can mediated the ERα signaling, ERα signaling can also alter the genomic distribution of the FOXA1 (Hurtado et al., 2011; Swinstead et al., 2016). Our previous work shows that the ERα chromatin recruitment in tamoxifen resistant cells is E2 dependent (Zhang et al., 2020), and this work indicates that the FOXA1 chromatin occupancy in tamoxifen resistant cells is also E2 dependent. The active FOXA1 transcriptional regulatory networks may facilitate the development of tamoxifen resistance. To determine whether the FOXA1 cistromes synchronize the transcriptional activity in tamoxifen resistance, we integratively analyze the FOXA1 ChIP-seq and RNA-seq data of MCF7 and MCF7/TamR cells. We find that the chromatin binding densities of FOXA1 and H3K27ac are enhanced around the TSS of the upregulated genes, and decreased around the TSS of the downregulated genes in MCF7/TamR cells. The transcription regulation and transcriptional activity exerted by FOXA1 may play an important role in the tamoxifen resistance. Previous reports show that FOXA1 can employ super enhancers through enhancement of H3K27ac modification to synchronize transcriptional reprogramming in tamoxifen resistance (Fu et al., 2019). We also find that FOXA1 is active in the transcription regulation in the enhancer regions, and further identify 1126 super enhancers in MCF7/TamR. As there are large numbers of lncRNAs and they extensively participate in the occurrence and development of cancers, we aim to investigate the super enhancer associated lncRNAs in tamoxifen resistance. We get five super enhancer associated lncRNAs, and finally identify one lncRNA ATP1A1−AS1 that may work as prognosis marker in tamoxifen resistance. ATP1A1−AS1 is an antisense transcript of ATP1A1, which is also an enhancer associated transcript, and they are positively correlated with each other. According to the lncRNAs working mechanism (Schmitt and Chang, 2016), ATP1A1-AS1 and ATP1A1 may positively promote the transcription activity or the stability of their transcript, which need further study. ATP1A1-AS1 may play an important role in tamoxifen resistance.

## Conclusion

Our work presents the FOXA1 differential cistromes, and they may synchronize transcriptional reprogramming in tamoxifen resistance. We also find the FOXA1 chromatin recruitment events in tamoxifen resistance may be also E2-dependent as ERα, and the specific mechanisms remain further study. We finally get one super enhancer associated lncRNA ATP1A1-AS1 that may work as biomarker or therapeutic target of tamoxifen resistance.

## Data Availability

The original contributions presented in the study are included in the article/Supplementary Material, further inquiries can be directed to the corresponding authors.

## Supplementary material

Supplementary Figure S1 The FOXA1 **(A,B)** and H3K27ac **(C,D)** chromatin binding densities plot of the non-differentially regulated genes (-1<log2Foldchange<1 & FDR>0.05) in MCF7 and MCF7/TamR cells, red line represents MCF7/TamR cells and the blue line represents MCF7 cells.

Supplementary Figure S2 Potential function of FOXA1 and H3K27ac in MCF7/TamR. BETA analysis of the FOXA1 **(A)** and H3K27ac **(B)** binding and expression data sets from MCF7/TamR cells. Red line represents up-regulated genes, purple line represents down-regulated genes, and dashed line represents the non-differentially-expressed genes as background.

Supplementary Figure S3 The KM survival curve of CASC15 **(A)**, KCTD21−AS1 **(B)**, and LINC00885 **(C)** using the luminal breast cancer treated with endocrine therapy datasets.
