# Supplementary figures and images for: Genome-wide analysis of the FOXA1 transcriptional regulatory network identifies super enhancer associated LncRNAs in tamoxifen resistance
Source: Front Genet. 2022 Sep 20;13:992444. doi: 10.3389/fgene.2022.992444 (PMC9530462; doi:10.3389/fgene.2022.992444)

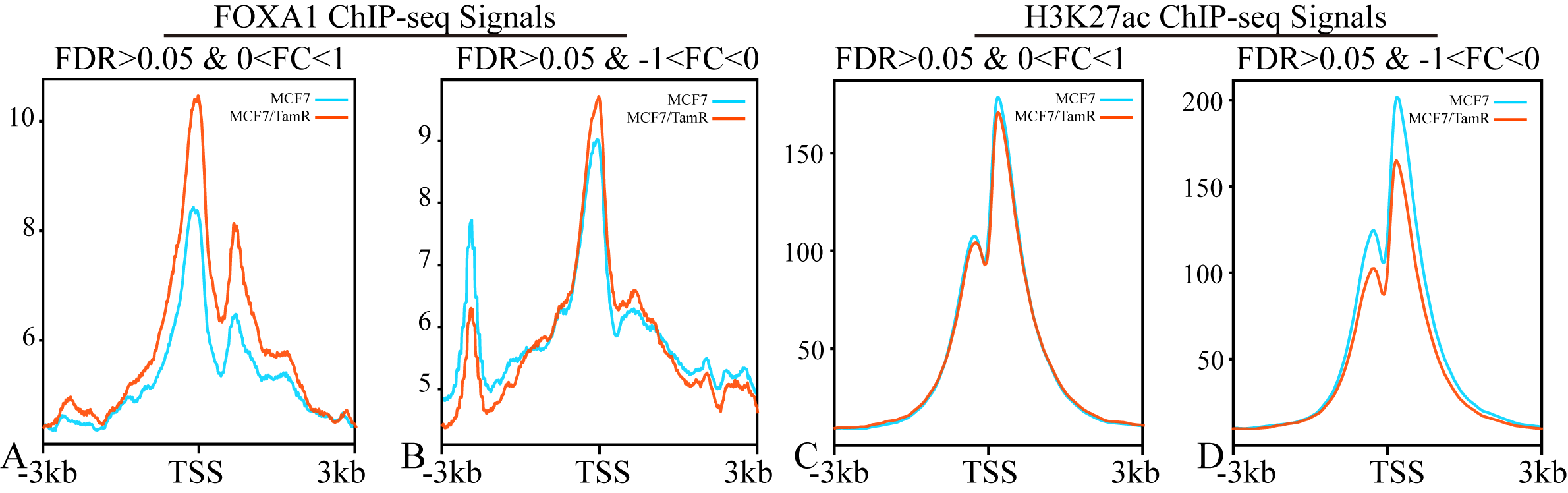

Supplement: Supplementary file 3 [file Presentation1.zip › Suppl. Figure 1.TIF]

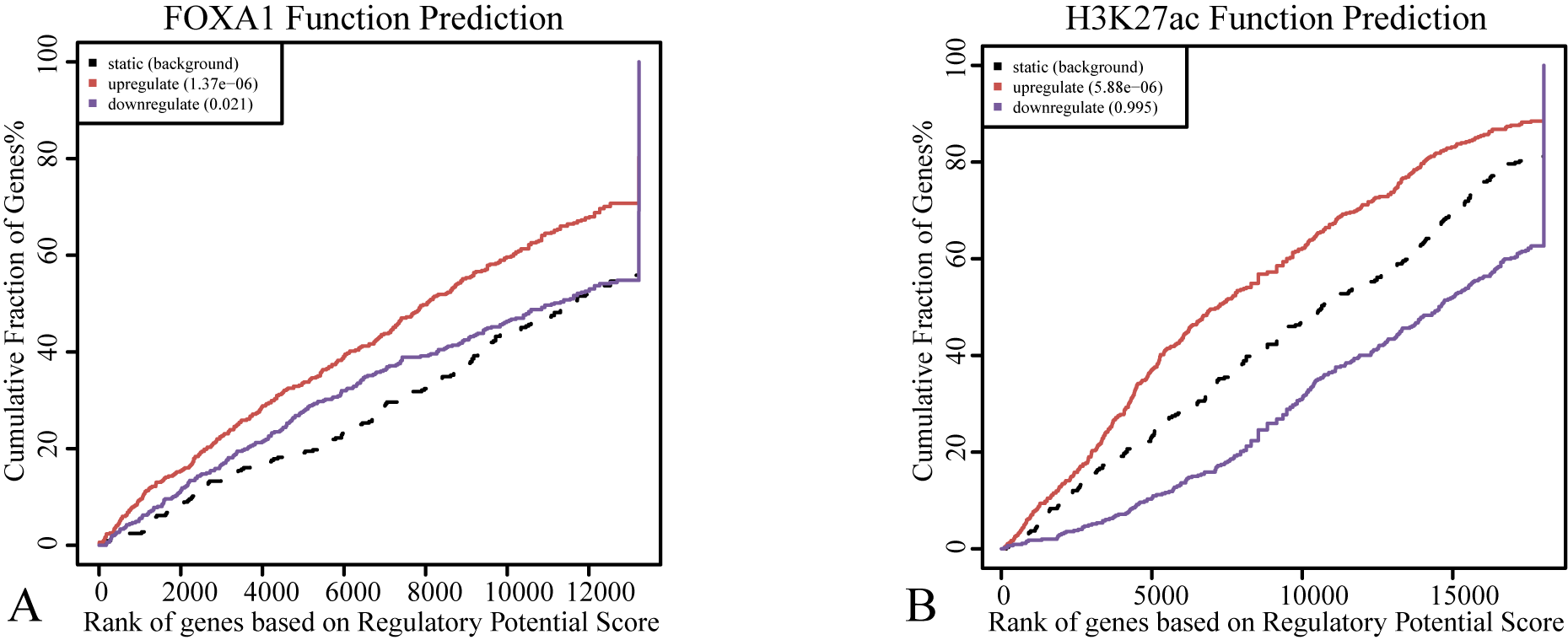

Supplement: Supplementary file 3 [file Presentation1.zip › Suppl. Figure 2.TIF]

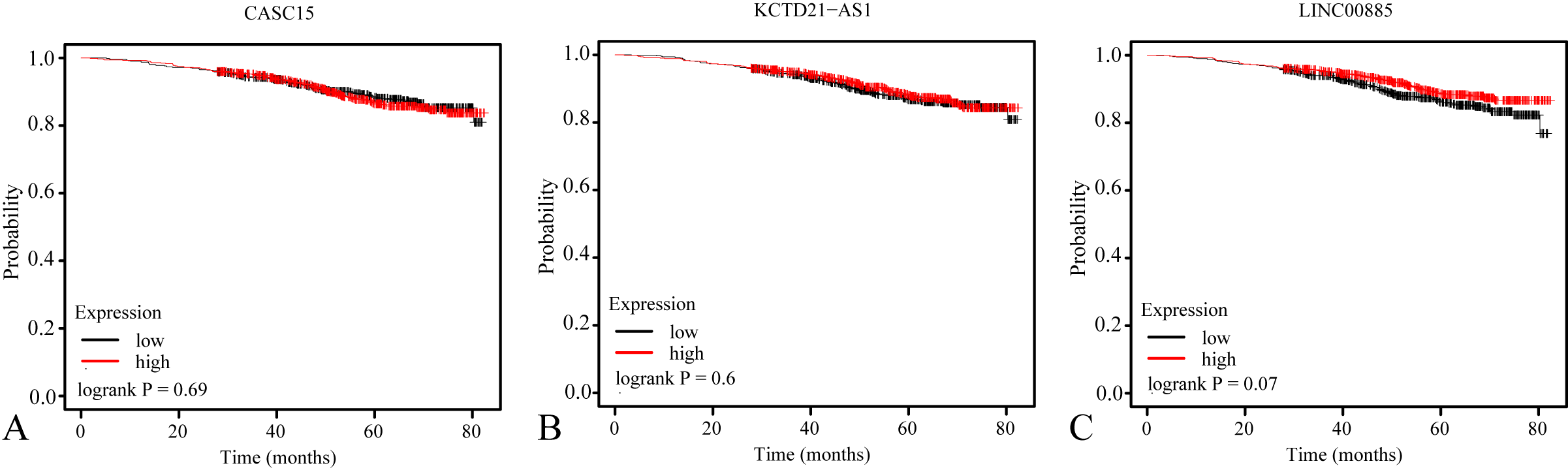

Supplement: Supplementary file 3 [file Presentation1.zip › Suppl. Figure 3.TIF]
